# Amalgam Fillings

**Published:** 1873-11

**Authors:** Thomas Fletcher


### ARTICLE VII.

### *Amalgam Fillings.*

BY THOMAS FLETCHER, ESQ., F. C. S.

The series of experiments which have now been carried on for a considerable time have brought prominently forward one serious fault existing in a large number of amalgams, to which sufficient attention has not been paid, and to which a great number of failures may be attributed, possibly more than may be fairly referred to shrinkage. This fault is a strong tendency to assume a spheroidal form whilst soft, after the filling has been inserted, resulting in a rounding of corners, leaving frequently a considerable space in the most dangerous and unexpected places. As may be imagined this occurs to the greatest extent when the amalgam is used very soft, and the use of glass tubes of sharp triangular forms shows this fault with many amalgams in a most glaring manner, although they may be practically free from absolute shrinkage of bulk. In the glass-tube test suggested by me some twelve months ago it will be found that many amalgams apparently shrink near the surface in a well-defined ring, which only extends a short distance down. As this, from its purely local character, cannot be entirely attributed to shrinkage, a series of experiments was instituted with sharp angular tubes, which at once proved that the greater part of the fault was caused by the alteration of form and tendency to round off any angles in which the amalgam had been packed. The round tube test, therefore, although a trustworthy guide in the choice of amalgams, does not give what may be called the spheroidal test in such a clear, distinct manner as sharp angular ones, which should be taken in preference in cases of exact experiment for comparative value.

The power of an amalgam to lie absolutely dead in position when packed is, so far as my experiments go, much more important than the question of moderate shrinkage, and one to which special attention should be paid, any fault in this respect resulting in the immediate penetration of fluids down, by any angles, to the bottom of the cavity and the immediate lifting of the plug by capillary force. An amalgam of this kind can, therefore, never make a solid, firm plug, and its existence as a protection is short, whatever value it may have as regards absence of shrinkage. It appears to me that the moisture which immediately penetrates under the plug by capillary action worked backwards and forwards by the intermittent pressure in mastication, and that the plug can never be steady and firm from the day it was inserted. This explanation would account for the appearance of a cavity from which an imperfect amalgam plug has been removed, the decay having progressed to the greatest extent at any angles in the cavity, extending more or less over the whole of the surface. If the failure had been caused entirely by shrinkage the decay would have progressed equally all over the surface, which is by no means the case where an amalgam of this class has been used.

The defect may be partially remedied in the most imperfect preparations by using them as free from mercury as possible, by thorough condensation in the angles and narrower parts of the cavity, and by a careful, firm burnishing before they became thoroughly hard. They cannot, however, be depended on as, whilst soft, the amalgam springs back when the pressure of the burnisher is removed and the moisture penetrates immediately; in this case no amount of after pressure will remove it, and a few years afterwards comes the end of the filling, and, unfortunately, often the end of the tooth also.—*British Journal of Dental Science.*

---

